# Development of Wearable Wireless Multichannel f-NIRS System to Evaluate Activities

**DOI:** 10.3390/mi16050576

**Published:** 2025-05-14

**Authors:** Xiaojie Ma, Tianchao Miao, Fawen Xie, Jieyu Zhang, Lulu Zheng, Xiang Liu, Hangrui Hai

**Affiliations:** 1 School of Mechanical Engineering, Henan Institute of Technology, Xinxiang 453003, China; maxiaojie@hait.edu.cn (X.M.); zhenglulu@hait.edu.cn (L.Z.); 15518082631@163.com (X.L.); 15194506295@163.com (H.H.); 2 School of Computer Science and Technology, Henan Institute of Technology, Xinxiang 453003, China; miao.csts@hait.edu.cn; 3 State Key Laboratory of Robotics and Systems, Harbin Institute of Technology Shenzhen, Shenzhen 518055, China; 25b363003@stu.hit.edu.cn

**Keywords:** fNIRS, multichannel, mental algorithm

## Abstract

Functional near-infrared spectroscopy is a noninvasive neuroimaging technique that uses optical signals to monitor subtle changes in hemoglobin concentrations within the superficial tissue of the human body. This technology has widespread applications in long-term brain–computer interface monitoring within both traditional medical domains and, increasingly, domestic settings. The popularity of this approach lies in the fact that new single-channel brain oxygen sensors can be used in a variety of scenarios. Given the diverse sensor structure requirements across applications and numerous approaches to data acquisition, the accurate extraction of comprehensive brain activity information requires a multichannel near-infrared system. This study proposes a novel distributed multichannel near-infrared system that integrates two near-infrared light emissions at differing wavelengths (660 nm, 850 nm) with a photoelectric receiver. This substantially improves the accuracy of regional signal sampling. Through a basic long-time mental arithmetic paradigm, we demonstrate that the accompanying algorithm supports offline analysis and is sufficiently versatile for diverse scenarios relevant to the system’s functionality.

## 1. Introduction

Certain individuals, particularly those who operate high-precision instruments, must maintain a high level of concentration for extended periods. Any decrease in attention may potentially lead to instrument damage and could even pose a risk to the operator’s physical wellbeing [1]. The psychological characteristics of individuals can reveal their attentional state throughout the duration of a given task. Brain–computer interface (BCI) technology, which has experienced rapid advances in recent years, represents a highly effective means of assessing an individual’s attentional characteristics [2,3].

In the 1990s, a seminal era began in the field of near-infrared spectroscopy (NIRS), sparking a proliferation of efforts aimed at creating functional NIRS (fNIRS) systems tailored for various applications [4,5,6]. The distinctive spatiotemporal properties of fNIRS have led to its emergence as one of the most popular BCI methods in nonmedical settings. As a noninvasive neuroimaging technique, fNIRS employs numerous acquisition devices with varying levels of precision in different application scenarios [7]. The collected signals are frequently used for longitudinal monitoring, which involves the measurement of changes in light intensity within tissue using light sources and detectors. The intensity signals captured by the sensors are typically employed to estimate the concentrations of oxyhemoglobin (HbO) and deoxyhemoglobin (Hb) through the application of the modified Beer–Lambert law [8,9,10,11].

Noninvasive BCI technology is frequently used for rehabilitation therapy following illnesses, particularly in facilitating the recovery of cognitive abilities after stroke surgery and maintaining cognitive function in individuals suffering from Alzheimer’s disease [12]. BCI technology primarily serves as an auxiliary tool in the therapeutic process, aiding medical professionals in evaluating the cognitive capabilities of patients. Among the commonly employed techniques are electroencephalography (EEG) and fNIRS, both of which are characterized by their adherence to the 10–20 international standard electrode placement system. EEG devices consistently conform to this standard, and it has been adopted by a significant number of fNIRS systems. However, compared with EEG devices, the application of the 10–20 international standard in fNIRS equipment poses several challenges, particularly in terms of patient positioning and placement of rod-shaped optodes [13].

During the treatment process, patient activities are frequently restricted when wearing equipment, hindering the implementation of numerous commonly used assessment paradigms. Eastmond et al. [14] argued that the fNIRS technology has a deep spatial correlation, which constitutes a notable difference from EEG. Furthermore, the temporal correlation curve of fNIRS signals exhibits smooth variations because of the inherent physiological characteristics of hemoglobin [15]. Conversely, the temporal curve exhibited by EEG signals is discrete across the time domain due to the inherent characteristics of electrical signals. Thus, for fNIRS technology, it is imperative to prioritize the investigation of distinct cognitive paradigms involving various regions of the brain. Data collection can be conducted in those regions of the brain that are relevant to the assessment.

Extensive research has investigated the responses of distinct brain regions to cognitive paradigms. In 1884, a theory derived from the examination of human brain regions and anatomical comparisons with the brain of other animals emerged, stating that the prefrontal cortex (PFC) is the primary region responsible for high-level cognitive functions [16]. This conjecture was subsequently validated by Brodmann through analysis of tissue structure and functional connectivity, leading to a more nuanced classification known as Brodmann’s area [17]. According to this classification, the brain’s two hemispheres are primarily segmented into the frontal, parietal, occipital, and temporal lobes.

In a noteworthy study exploring the sustained attention of offshore workers, Fan and Blanco-Davis asserted that the PFC exhibits sensitivity towards task execution and decision-making capabilities [18]. Prior to that, Xiao et al. had conducted a study on regional brain activities among children diagnosed with high-functioning autism and attention deficit hyperactivity disorder [19]. Using a 16-channel fNIRS device, they collected physiological signals from the PFC, corroborating the presence of elevated levels of inactivation within the PFC of these patients. Building upon this research, Ikeda et al. captured signals from the temporal lobe through the addition of extra channels, and found that signals originating from the frontal and temporal lobes can serve as viable indicators for real-time attention monitoring [20]. However, the signal response from the temporal lobe is relatively subdued. Furthermore, numerous studies have emphasized the ability of the commonly employed mental arithmetic (MA) paradigm to elicit distinct and robust activation signals within the frontal lobe region [21]. Given these considerations, the present study employs the MA paradigm.

To better understand the responses of the PFC during cognitive activities, Bracken et al. developed a portable and robust single-channel wireless fNIRS sensor. This innovative system facilitated the comparison and analysis of responses from the PFC across tasks of varying intensity. However, the practical application of such systems is often challenged by excessive weight, which leads to a significant increase in motion artifacts. In response to this challenge, the research team incorporated an acceleration sensing module into the device to quantitatively assess motion artifacts [22]. This enhancement increased the functionality of the system, but also contributed to an increase in the size and weight of the sensor, resulting in higher manufacturing costs.

To address these concerns, a multichannel wireless fNIRS system tailored for PFC measurements is developed. This advanced system reduces the size and weight of the sensor without compromising data accuracy or exacerbating artifact levels. Such refinements ensure improved ease and comfort of wear, thus optimizing the system’s utility in practical applications. This article describes the development of this wireless multichannel fNIRS system using two light sources and a detector. The relevant algorithms are incorporated into the hardware to increase the reliability of data collected from the PFC. This paper is organized as follows. Section 4 provides an in-depth exposition of the hardware design; Section 3 evaluates the stability of the system through performance testing experiments conducted on the designed sensor and employs the MA paradigm to measure the system resolution; Section 4 discusses the comparison of the experimental results of the MA paradigm with the capabilities of existing research and finally encapsulates the research trajectory outlined in this article.

## 2. System Design and Methods

In a review of current functional near-infrared products and relevant studies, we found a common trend in the wavelengths emitted by transmitters, typically in the range of 650–850 nm [23]. Based on the principles of hemodynamics, it is well known that hemoglobin has favorable scattering properties for near-infrared light within the wavelength spectrum of 650–900 nm. Specifically, both HbO and Hb exhibit higher absorption rates for near-infrared spectra in this wavelength range, while other components exhibit comparatively lower absorption rates [24]. Thus, we use two near-infrared wavelengths of 680 nm and 850 nm.

**Figure 1:**
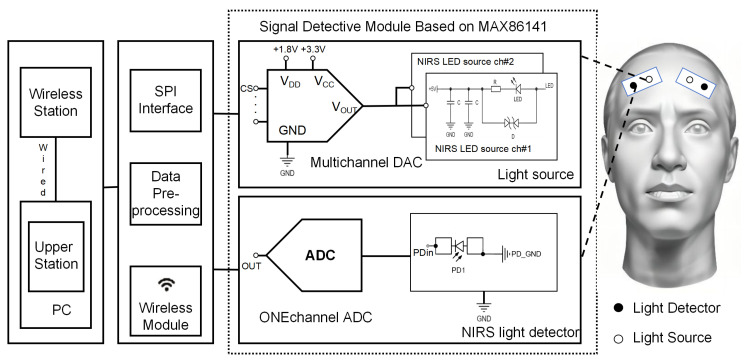
Our proposed fNIRS system, which includes the sensor part, wireless base station, and upper part, is presented.

Figure 1 illustrates the proposed system. There are two distributed sensor parts to capture variations in brain blood oxygen signals associated with PFC activity. To capture multichannel signals from the PFC, we must ensure that the sensors meet the specific usage requirements. Each part consists of a two-channel light source module and a single-channel photodiode (PD) light detector module, both controlled by the MAX86141 chip (Maxim Integrated Inc., San Jose, CA, USA). Furthermore, an MCU acts as the central controller within each component, along with a built-in wireless communication module for seamless data transmission. When positioned on the forehead, the light source probe and photodetector probe enable light emission and collection, respectively. The collected light signals undergo analog-to-digital conversion (ADC) before transmission to the MCU via the serial peripheral interface (SPI) protocol embedded within the module.

The MCU then performs preliminary preprocessing on the data before wireless transmission to a designated wireless base station. This station serves as a central hub, collecting data from all sensor parts and transmitting them to a host computer via a wired protocol for further visualization and processing.

### 2.1. Hardware of fNIRS System

**Figure 2:**
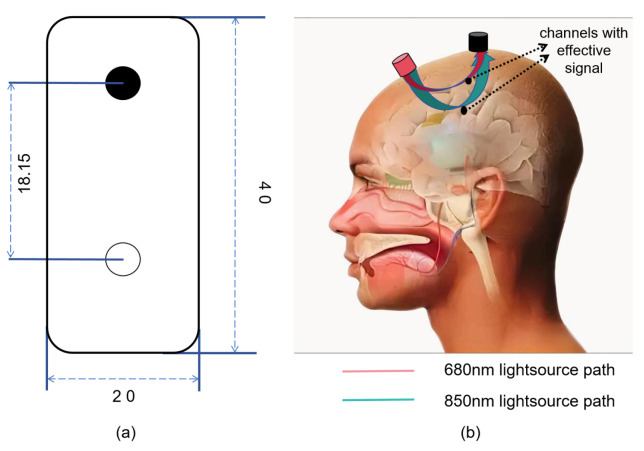
Schematic diagram of the sensor analog device placement and the near-infrared light path during signal acquisition. (**a**) Sensor Backside Design Diagram. (**b**) The optical path of near-infrared light emitted by the emitter and detected by the detector at different wavelengths within the intracranial space.

The sensor component of our system uses the MAX86141 chip, which can handle three fNIRS light sources and a pair of fNIRS detectors. We use two near-infrared wavelength channels and a single fNIRS detector based on the sensor dimensions. Specifically, we use a dual-wavelength LED emitter with wavelengths of 680 nm and 850 nm. The detectors are positioned 18.15 mm from one another, as shown in Figure 2, and are aligned with the prefrontal region to collect sensor data. Figure 2a shows the distribution of emitters and receivers on the sensor board. The different refractive indices in various mediums mean that the optical paths of the emitted light will differ after passing through the skull and tissue fluid, allowing the capture of oxygen information from blood at different depths, as shown in Figure 2b. The MAX86141 offers integrated AD/DAC functionality, enabling direct control of the optical emitter and receiver. This facilitates the acquisition of quantified light intensity signals. The FIFO cache of this chip stores light intensity data sequentially as $I^{\lambda_{1}},I^{\lambda_{2}}$, where *I* and $\lambda_{j}$ represent the PD detector that receives light at wavelength $\lambda_{j}$. The pulse width is set to 129.8 μs and the sampling frequency is 512 Hz. To downsample the data and reduce noise, the average over 32 contiguous samples per channel is calculated, resulting in an effective sampling frequency of 16 Hz.

### 2.2. Data Acquisition with Dual-NIRS Light

The signal data captured by the MAX86141 chip are fed into the NRF52832 (NORDIC Semiconductor Inc., Trondheim, Norway) via the short-range SPI protocol. Our approach involves streamlining the sensor architecture, using the NRF52832 for fundamental data preprocessing tasks such as digital signal reordering and temporal alignment. Furthermore, the 2.4 G wireless transmission capability of the NRF52832 is harnessed to relay digital signals to the base station using a ceramic ANT3216 antenna. This design streamlines the peripheral circuit size of the sensor while mitigating potential electromagnetic interference issues that may arise in the absence of electromagnetic shielding. Wireless distance assessments have demonstrated that the communication range between the sensor module and the base station can extend up to 8 m, which is adequate for our intended applications.

In the realm of wireless transmission systems, power consumption is a crucial metric. To ensure a user-friendly system, we opt for a small 280 mAh lithium battery to drive each sensor component. Leveraging the minimal power demands of the MAX86141 and our fine-tuning of the NRF52832 for low-power mode operation, a single sensor can sustain continuous data collection for up to 4.5 h. Given the varying voltage requirements across sensors, in addition to the essential lithium battery charging and discharging protection circuitry, we devise a DCDC voltage boost and buck circuit to power our chips. This DCDC circuit comprises a 5 V boost segment, a 3.3 V buck segment, and a 1.8 V buck segment. To mitigate power losses during voltage regulation processes, we employ the TPS61022 chip (Texas Instruments Inc., Dallas, TX, USA) for enhanced operational efficiency.

Owing to the scattering properties exhibited by conventional near-infrared light within biological media, and in accordance with hemodynamic principles, photons emitted by near-infrared light sources undergo significant scattering and reflection as they traverse through tissue and blood. A fraction of these photons are reflected back from the cerebral cortex [25]. Concurrently, the barrier posed by the skull means that photons of higher intensity are reflected from subcutaneous capillaries, whereas those of lower intensity are reflected from brain tissue fluid after penetrating the skull. The diffuse paths of photon reflection and transmission detected by photodiodes within brain tissue, spanning from the source to the detector, manifest a banana-shaped pattern.

The modified Beer–Lambert law effectively elucidates the correlation between light intensity and solution concentration [10], enabling the monitoring of variations in light intensity in human tissue for the assessment of light attenuation. With the low power of the emission source, human tissue and blood can be regarded as a homogeneous mixed solution, maintaining a constant absorption coefficient for light in tissue. The sensor chip captures light intensity signals, facilitating the conversion of these signals into concentration data for two types of hemoglobin in the NRF chip.

(1) $$\Delta OD=\ln\frac{I_{0}^{\lambda }}{I_{\det}^{\lambda }}=L\cdot \Delta \mu_{\varepsilon }+G^{\lambda }$$

where ΔOD denotes the change in optical density, indicative of light attenuation within tissue; $I_{0}$ and $I_{det}$ represent the incident and detected light intensities of tissue under different conditions, which are crucial parameters in quantifying light attenuation; *L* is the overall average path length of the detected photons, which dictates the propagation distance of light in tissue; $\mu_{\varepsilon }$ denotes the absorption coefficient of the tissue, illustrating its light absorption capacity; and *G* represents the effect of tissue light absorption. This latter parameter is subject to variations in optical pathways, making precise determination challenging. Our presumed model simplifies the issue by treating the tissue fluid as a uniform mixed solution. Here, *L* is correlated with the differential path length factor DPF and the source–detector separation distance *d*. DPF is a crucial parameter delineating the scattering and path deviation traits of photons in tissue, while *d* directly dictates the light’s propagation distance in tissue:

(2) L=DPF·d

Changes in near-infrared light attenuation are directly proportional to alterations in absorption, indicating the comprehensive impact of variations in the concentration of Hb and HbO (denoted as $C_{Hb}$ and $C_{HbO}$, respectively). During this process, the absorption coefficients of the various chromophores are quantified using weights ε, which collectively influence the degree of light attenuation within the tissue.

(3) $$\Delta \mu_{\varepsilon }=\varepsilon_{Hb}^{\lambda }\cdot \Delta C_{Hb}+\varepsilon_{HbO}^{\lambda }\cdot \Delta C_{HbO}$$

In Equation (1), the value of *G* is indeterminate due to its variability between different measurement positions. Nevertheless, within a single measurement, *G* can be approximated as a constant. Instead of focusing on specific concentrations of HbO and Hb at a given instant, our primary interest is the relative alterations in hemoglobin concentrations throughout the test procedure. Therefore, by comparing the optical values at times *t* and $t_{0}$, we can derive the relative changes in $C_{Hb}$ and $C_{HbO}$, which are adequate for our purposes. Precise knowledge of the exact value of *G* is not essential for analyzing dynamic fluctuations in hemoglobin concentrations.

(4) $$\Delta {OD}^{\lambda }={OD}^{\lambda }\left(t\right)-{OD}^{\lambda }\left(t_{0}\right)=\ln\frac{I_{1}^{\lambda }\left(t\right)}{I_{2}^{\lambda }\left(t\right)}-\ln\frac{I_{1}^{\lambda }\left(t_{0}\right)}{I_{2}^{\lambda }\left(t_{0}\right)}$$

(5) $$\Delta {OD}^{\lambda }=\Delta \mu_{\varepsilon }\cdot \Delta L\cdot DPF$$

In Equations (3) and (5), $\Delta \mu_{\varepsilon }$ depends on $\varepsilon_{Hb}$ and $\varepsilon_{HbO}$. The numerical values of these parameters can be found in [26], while the value of DPF is given in [27]. Considering that the relative concentrations of Hb and HbO are not specified, a binary equation can be constructed using two near-infrared light wavelengths. Solving this matrix equation makes it possible to determine the relative concentration of oxygen in the blood at a specific time. The matrix transformation process is as follows:

(6) $$\begin{array}{cc} \; & \left[\begin{array}{c} \Delta {OD}^{680}\\ \Delta {OD}^{850} \end{array}\right]\left.=\left[\begin{array}{cc} \varepsilon_{Hb}^{680}\cdot {DPF}^{680}\cdot \Delta L & \varepsilon_{HbO}^{680}\cdot {DPF}^{680}\cdot \Delta L\\ \varepsilon_{Hb}^{850}\cdot {DPF}^{850}\cdot \Delta L & \varepsilon_{HbO}^{850}\cdot {DPF}^{850}\cdot \Delta L \end{array}\right.\right]\cdot \left[\begin{array}{c} \Delta Hb\\ \Delta HbO \end{array}\right] \end{array}$$

(7) Y=F·X

The optical density vector *Y* contains the measured light attenuation data, and *X* represents the relative hemoglobin concentration values. Using least-squares analysis [28], we can derive a constant matrix *K* linked to matrix *F*, which in turn is associated with another constant matrix *F*.

(8) X=K·Y

(9) $$K={\left(F^{T}\cdot F\right)}^{-1}\cdot F^{T}$$

Given that *Y* and *K* are both constant matrices, the relative concentration matrix *X* can be determined from Equation (8).

### 2.3. System Implementation

**Figure 3:**
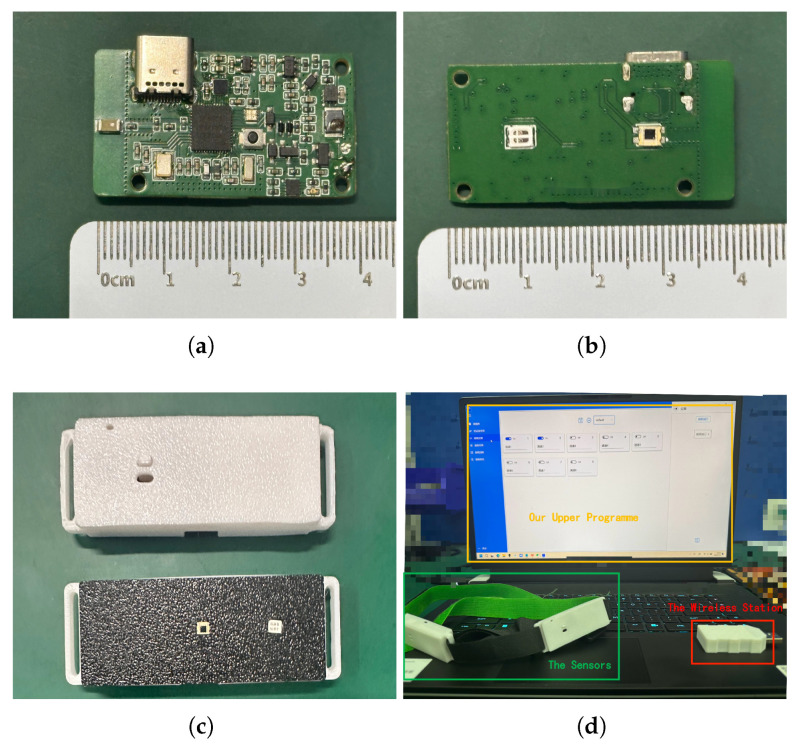
Implementation of multichannel fNIRS system hardware platform. (**a**) Top of fNIRS sensor PCB. (**b**) Bottom of fNIRS sensor PCB. (**c**) Shell of NIRS sensor PCB. (**d**) Complete schematic diagram of fNIRS sensor system.

The final implementation of the multichannel wireless fNIRS system is illustrated in Figure 3, which shows its hardware components. In terms of the fNIRS sensors, the PCB dimensions are set at 40 × 20 mm, as outlined in Figure 3a. The light source emitter and receiver are meticulously arranged on the back of the PCB, maintaining a precise distance of 18 mm to enhance measurement accuracy, as shown in Figure 3b.

For hardware protection and stability, the casing is 3D printed using polylactic acid, with dimensions of 40 × 20 × 10 mm. The casing features a USB-C interface for convenient sensor charging and snap-fit elements on both sides for easy attachment to the head, ensuring comfortable wear and use, as illustrated in Figure 3c.

Figure 3d provides a comprehensive view of the different components of our fNIRS system, which includes distributed multichannel sensors, a wireless receiver base station, and host software. The wireless base station connects to the host software using the USB protocol, enabling stable and efficient data transmission.

In tackling the ubiquitous challenge of the temporal alignment of data within distributed sensor systems, various methods have been employed. By incorporating the recording and zeroing functionalities into the upper computer design, establishing the initial time $t_{0}$, and synchronizing all sensors with a unified timestamp, temporal consistency of data collection is successfully achieved. This approach effectively mitigates time offsets during data acquisition and resolves synchronization discrepancies among various sensors.

## 3. Experiments and Results

### 3.1. System Reliability Verification and SNR Testing

First, given the sensitivity of fNIRS sensors to ambient light, even minor fluctuations in ambient light levels can result in a significant decrease in the signal-to-noise ratio (SNR). Therefore, accurate measurements of the ambient light intensity are crucial in validating the reliability of the sensor. These measurements help ensure that the overall oxygenation signal is accurate and is not compromised by external lighting conditions.

**Figure 4:**
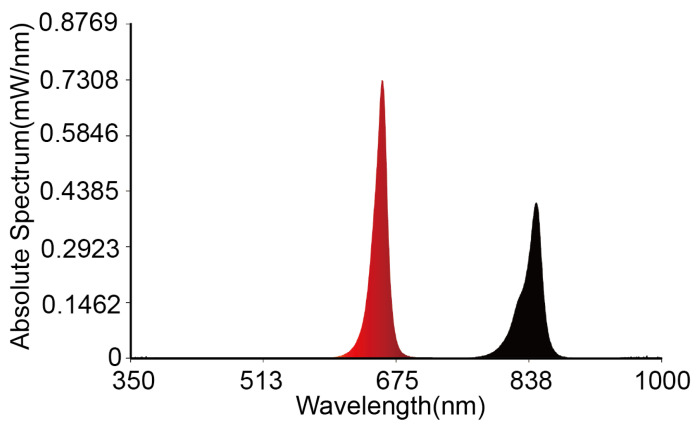
The results of the emitted light spectrum tests. The red and black components represent 650 nm and 850 nm, respectively.

We first tested the infrared light spectrum emitted by the emitter to ensure the accuracy of data calculations in the formula. As shown in Figure 4, the main wavelength is 642.2 nm, with a peak wavelength of 658.2 nm for the red portion and a color purity of 99%, and a half-width of 17.0 nm. The auxiliary wave’s peak wavelength for the black part is 846.3 nm, with a half-width of 22.6 nm. These values meet the requirements for sensor calibration.

Before testing the performance of the proposed system, specific adjustments were made to the MAX86141 sensor settings. By activating the ambient light compensation circuit (ALCC), a key feature within the MAX86141 chip that helps filter out ambient light at the hardware level, we facilitated the collection of a purer near-infrared signal. The receiver of the sensor was able to directly detect the ambient light intensity in the experimental environment. Subsequently, a signal conversion process was performed to allow the raw light intensity signals to be further analyzed and compared.

To ensure data accuracy, the sensor began recording data 2 min after preheating. Initially, the test personnel wore the sensor and remained in complete darkness for 7 min to measure the ambient light intensity under dark conditions and establish a baseline. Subsequently, to simulate more realistic scenarios, fNIRS sensors collected static data in normal environments for 7 min. This enabled a comparison of how the measured ambient light intensities in these two different environments would affect the overall environmental light noise while the device was in use.

**Figure 5:**
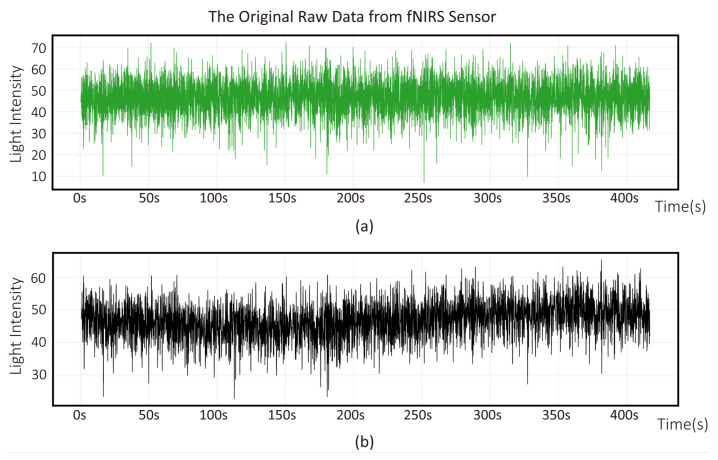
Process of converting the initial light intensity signal into hemoglobin concentration using the fNIRS system while the subjects are in a relaxed state. Variation of 7 min light intensity measured by fNIRS sensors in two environments. The upper figure (**a**) represents the normal environment and the lower figure (**b**) depicts the dark environment.

**Figure 6:**
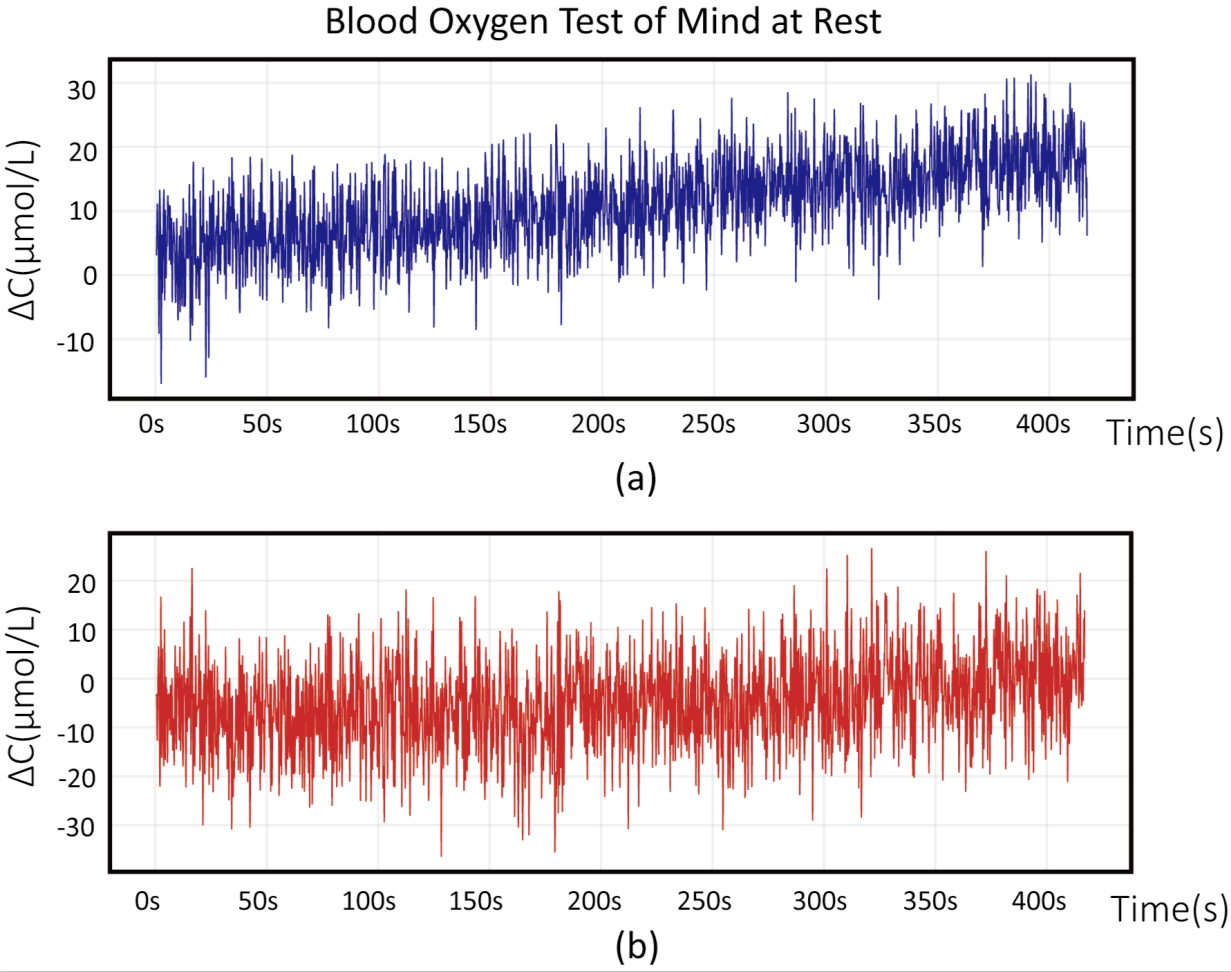
Process of converting the initial light intensity signal into hemoglobin concentration using the fNIRS system while the subjects are in a relaxed state in the normal room environment. The upper figure (**a**) represents $\Delta C_{Hb}$ and the lower figure (**b**) depicts $\Delta C_{HbO}$.

During clinical analysis using the device, as the lighting conditions in the room where cognitive training took place were similar to those in other ordinary rooms, we conducted tests by turning on all the lights in a 10-square-meter room. To minimize discomfort to the subjects during prolonged immobility of their heads and potential motion artifacts, sensors were placed on the forehead regions. The recording duration was chosen to minimize discomfort and ensure data integrity. Figure 5 and Figure 6 illustrate fNIRS signals and changes in the concentrations of Hb and HbO. Figure 6 shows the near-infrared light intensity signals detected by photodiodes, as calculated from two wavelengths according to the algorithm described in Section 2. Figure 5 shows the changes in the concentrations of Hb and HbO derived from Equation (8).

### 3.2. Data Acquisition with Dual-NIRS Light

The data under normal conditions match the baseline measured in the dark state, indicating that environmental light does not interfere with the signals. Additionally, the spikes in the blood oxygen concentration signal conversion curve highlight the high sensitivity of the sensor to minor fluctuations. This underscores the importance of keeping the subject immobile, as even slight head movements can introduce significant artifacts into the collected data. As is known, the head movements or muscle activities can affect the transmission of near-infrared light. This causes a change in the number of tissues through which light travels within the measurement area, leading to motion artifacts, i.e., other false physiological activity signals. Motion artifacts in the blood oxygen signals typically manifest as nonlinear signal jumps, which can complicate subsequent signal analysis. Therefore, efforts should be made to minimize the generation of motion artifacts. For those that do occur in the signal, we employed a special filter for their removal.

### 3.3. Bandage Masking Experiment

The fundamental principle of the fNIRS system revolves around the detection of blood oxygen information. To verify the precision of this system in measuring cerebral oxygen levels, we followed Cui et al. [29] in conducting a bandage masking experiment to illustrate consistent alterations in blood oxygen information. Six healthy subjects took part in this investigation. Table 1 outlines their basic details and pre-experiment blood pressure readings. Subject 0 used a commercially available blood oxygen sensor to facilitate a direct comparison of variations in the blood oxygen curve. Following data collection at the hardware level, we extracted the raw blood oxygen concentration signals from the host computer. To guarantee data precision, we devised a tailored filter to manage high-frequency noise and enhance the processing of the raw data.

**Figure 7:**
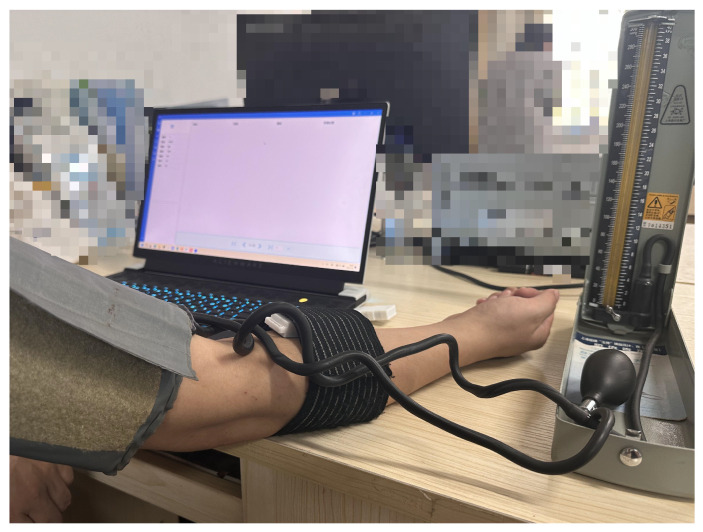
Bandage masking experiment: fNIRS sensors were strategically positioned over the dorsal surface of the front arm, while a blood pressure cuff was appropriately affixed to the upper arm.

**Figure 8:**
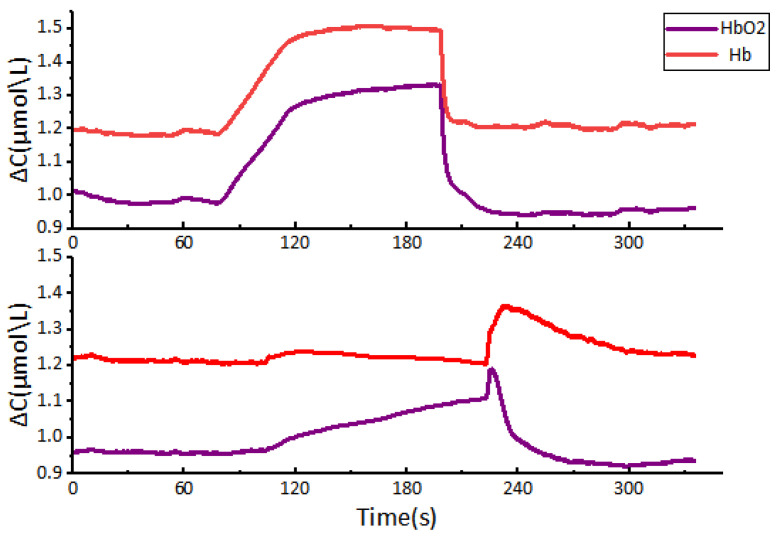
Subject 0 bandage masking experiment data using a commercial NIRS sensor system. The upper graph shows the changes in HbO and Hb measured at an applied pressure of 40 mmHg. The lower graph illustrates the trends in HbO and Hb measured at an applied pressure of 160 mmHg.

Figure 7 shows a participant during the bandage masking test. When the inflation pressure of the cuff reaches 40 mmHg, which is below the diastolic pressure, the venous blood flow is obstructed. This leads to the initial state of arterial blood flow during systole, a decrease in the velocity of the venous blood flow, and, consequently, an increase in the concentrations of Hb and HbO. Once the cuff is deflated, blood flow returns to normal and the concentrations of Hb and HbO return to their baseline levels. When the inflation pressure of the blood pressure cuff is increased to 160 mmHg, which is above the systolic pressure, both venous and arterial blood flow is blocked, causing the blockage of blood flow between the forearm and the upper arm of the subject. The oxygen consumption of the forearm muscles remains unchanged due to cuff inflation, resulting in an increase in Hb concentration and a stable decrease in HbO concentration. Subsequently, upon deflation of the cuff, blood re-enters the area and the concentration of HbO rapidly increases, then gradually decreases, ultimately returning to its baseline level; during this period, the concentration of Hb exhibits the opposite trend. There may be fluctuations in the curves at the moments of cuff inflation and deflation. These can be attributed to upper limb muscle activity induced by pressure changes, leading to motion artifacts. These motion artifacts have obvious characteristics, and are therefore not removed because they do not substantially affect the trend of changes in blood oxygen concentration. The experimental results for Subject 0, ascertained through a commercially available blood oxygen monitoring system, are depicted in Figure 8.

**Figure 9:**
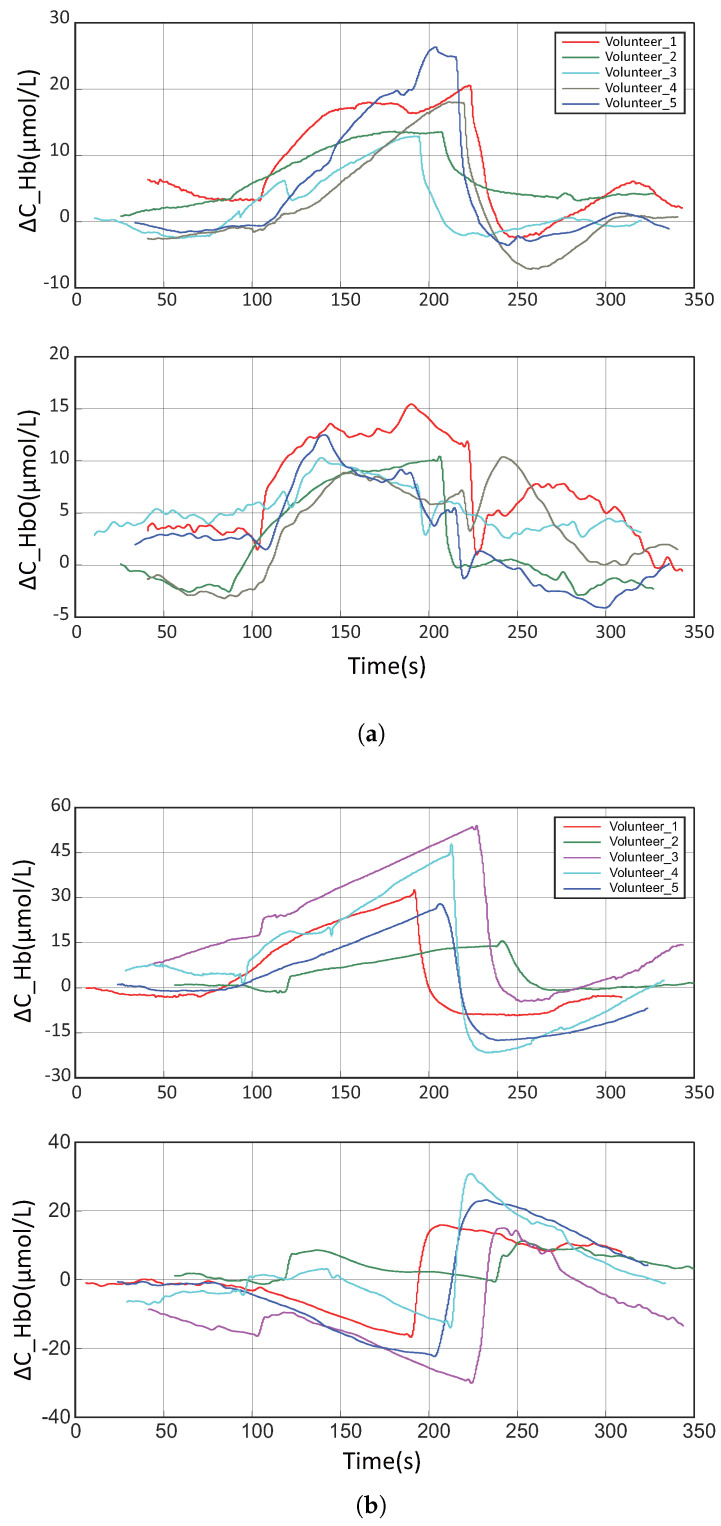
Experimental data curves of the bandage masking experiment. (**a**) Bandage masking experiment data under 40 mmHg pressure using our fNIRS sensor system. (**b**) Bandage masking experiment data under 160 mmHg pressure using our fNIRS sensor system.

To evaluate experimental accuracy, we calculated the Root Mean Square Error (RMSE) between datasets from five subjects and Subject 0. First, as our algorithm initializes the blood oxygen level at the sensor’s starting point as the zero baseline, negative values were observed in the raw data. To eliminate these artifacts, the zero point was adjusted to convert negative values to positive values, followed by a logarithmic transformation to harmonize the data with those obtained from the commercial sensor. Subsequently, 20 RMSE values were computed between the two datasets under these normalized conditions. The results demonstrated RMSE values ranging from approximately 0.0302 (minimum) to 0.1320 (maximum), with a mean of 0.09. These findings indicate that our sensor system produced blood oxygen variation curves that closely aligned with those of the commercial sensor during the experimental protocol. Figure 9a,b depict the data collected through our wireless system. Raw data obtained during acquisition reveal the presence of numerous motion artifacts. Prior to data processing, some temperature drift and subtle artifacts are unaddressed, leading to minor fluctuations in the curves. Nevertheless, these artifacts underscore the high precision of our sensor hardware in capturing near-infrared light. Furthermore, the general trend in changes in the blood oxygen signal corresponds to the data obtained by the commercial sensor system. The patterns in the variations of Hb and HbO align with the results reported by Cui et al. [29], validating the reliability of our wireless system in measuring blood oxygen activity.

### 3.4. MA Experiment

The results of the bandage masking tests successfully validated the reliability of our system. An MA experiment was then conducted to comprehensively evaluate the proposed fNIRS system. Basic MA experiments have gained universal recognition in the cognitive paradigm. These experiments enable the observation of significant consistent changes in blood oxygen indicators in the frontal lobe region [21].

Given the practical application scenarios, our system provides cognitive training benefits for users in their daily lives and offers reference data for rehabilitation therapists in medical settings. Therefore, real-time data detection and computation are not necessary; instead, evaluating the overall cognitive process at the end of the experiment is sufficient.

First, the paradigm for the MA experiment was designed. The commonly used MA paradigm involves switching between rest and calculation within a relatively short period of time. Considering the realistic cognitive scenario involving calculation and rest durations, we used a longer-duration MA experiment paradigm, called the long-time MA paradigm. This paradigm consists of a classic three-stage experimental process.

**Figure 10:**
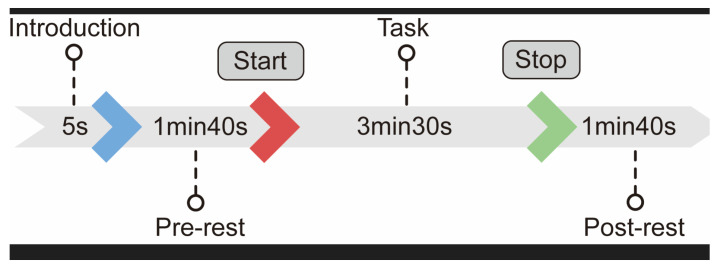
Cognitive attention experiment based on the MA paradigm. A 100 s rest period is followed by the completion of an arithmetic task over a period of 3 min 30 s, followed by another 100 s rest period.

The first stage is the resting state, which is used to determine the baseline level of blood oxygen parameters in the frontal lobe when the human body is in a calm state. The second stage is the calculation phase of the MA task, which lasts 3 min 30 s and simulates the cognitive training of MA in real-life scenarios. The final stage is another resting phase, which enables a comparison of the blood oxygen level during rest with the baseline level measured in the first stage. This phase also provides characteristic changes in blood oxygen levels after training, which helps identify moments of decreased attention during the training process. The overall MA paradigm is illustrated in Figure 10.

We invited five volunteers around the age of 23 to participate in this test. The subjects were in good physical health with no cognitive abnormalities. The test was conducted at approximately 09:00, when attention is expected to be high. The sensors were placed on the volunteers and the test began 10 s after an experiment briefing, with data recording starting simultaneously. After removing bad data and re-conducting the experiment collection, we obtained 20 sets of training data.

**Figure 11:**
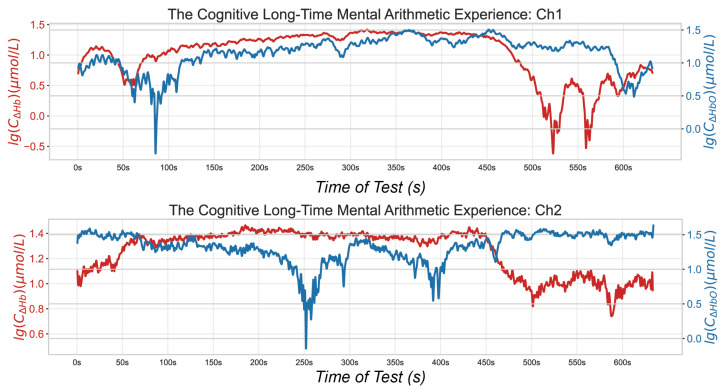
Oxygen signals obtained during MA tests: spectral density before and after processing in one volunteer.

Data processing and analysis were conducted after the data collection. First, the raw data were cleaned using a bandpass filter designed for blood oxygen signals and a baseline artifact removal algorithm [29,30]. The passband filter has a cutoff frequency range of 0.05 Hz to 0.4 Hz, effectively eliminating high-frequency noise from the instrument, physiological fluctuations, and significant baseline drifts due to temperature variations. Additionally, we incorporated the Time-Domain Deconvolution (TDDR) algorithm to address both slow baseline drifts and motion artifacts, enhancing data integrity because TDDR helps in mitigating subtle yet persistent baseline shifts and movement-related distortions, providing a more accurate and reliable signal for analysis. The data cleaning eliminated motion artifacts and baseline drift. Besides the signals of interest, the original signals contained physiological noise related to respiration and heartbeats, as well as high-frequency noise. The noise was minimized using the designed bandpass filter, as demonstrated in the signal power spectrum shown in Figure 11. After the cleaning process, the low-frequency blood oxygen signal power was retained and the remaining physiological high-frequency noise was effectively filtered out.

The curves already show significant changes in frontal lobe blood oxygen levels during MA tasks. Clearly, there are corresponding points of elevation and depression on the curve that align with the rest–activation transitions designed in the paradigm. Although individual differences mean that these transitions may occur at slightly different times, this variation is acceptable. Through the analysis of blood oxygen curves, we have determined that our sensor system is sufficiently sensitive to detect changes in blood oxygen levels in the dorsolateral PFC.

## 4. Discussion

In the realm of market-available brain oxygenation sensors, the predominant modality involves sampling from the frontal lobe with a single-channel configuration. Our research advances this by implementing a distributed array of multiple sensors, thereby augmenting the perceptive capabilities for monitoring human attention. Our exploration of NIRS sensors reveals that a dual-wavelength system suffices for the precision demands of these sensors. As indicated in the literature [31,32], even sensors that use a single detector of near-infrared light can, with appropriate signal processing, meet commercial performance benchmarks.

Upon determining several basic parameters, including SNR, we tested the sensor’s response to rapid changes in muscle oxygen saturation. This focus emerged from the understanding that cerebral blood oxygen variations are inherently slow, thereby limiting the assessment of the sensor’s proficiency in tracking blood oxygen concentration trends. Our analysis of blood oxygen saturation curves from six subjects revealed a subtle, but notable, rebound in oxygenated hemoglobin levels after a precipitous drop due to pressure release in all but one subject during a 40 mmHg pressure test. For Subject 0, who was monitored using a commercial apparatus, the upswing in the curve could be linked to inadvertent muscle micro-movements upon pressure dissipation, inducing an artifact.

Theoretically, according to physiological hemodynamics, there should be a decline in the concentrations of both deoxyhemoglobin ($C_{Hb}$) and oxyhemoglobin ($C_{HbO}$) after the removal of a pressure of 40 mmHg. We conducted a quantitative analysis comparing the data acquired by the gold-standard sensor and our sensor system during the experiments. Initially, we observed that although the tests were performed on the same muscle group, the collected blood oxygen data exhibited significant variations across different time points and among different subjects. This discrepancy can be attributed to inherent physiological characteristics of the human body. Of note is the fact that we acknowledged the significant individual physiological variances among subjects, wherein minor artifacts might be concealed amid the more pronounced oscillations characteristic of the cuff experiment. However, this unexpected increase was distinctly observable in the signal data. This phenomenon contrasts with the marginal increase detected immediately after relieving a pressure of 160 mmHg in Subject 0, which we infer to be motion artifacts and a muscle spasm in response to the sudden cessation of sustained pressure. Furthermore, the remaining five subjects demonstrated comparable fluctuations in blood oxygen levels under identical experimental conditions to Subject 0, corroborating the established hemodynamic principles.

In the MA test conducted in this study, we observed clear changes in blood oxygen levels corresponding to simple rest–activate–rest shifts in attention. We conducted detailed statistical analyses on the data, performing *t*-tests on three distinct stages of two-channel recordings from five subjects. The resulting two-tailed critical values for all *t*-tests were remarkably close to 1.96, with *p*-values below $10^{-6}$. This stringent threshold indicates a high level of confidence in the statistical significance between the data from the two channels. Table 2 and Table 3 show that the three key features of cerebral oxygenation changes during the two rest phases are largely consistent. However, there is a significant difference between the cerebral oxygenation changes and blood oxygenation changes during the training phase. Figure 11 illustrates the specific temporal changes in cerebral oxygenation across different stages. This effect can be largely attributed to the dorsolateral PFC, which shows changes in oxygen consumption and hemoglobin concentration during the transition between rest and activation states. Additionally, the two channels exhibited distinct patterns of blood oxygenation changes, potentially revealing different responses from left and right regions compared to the MA paradigm. This provides a multiregional reference for our subsequent algorithm research.

For our system, we selected the prefrontal cortex as the data collection area, considering the difficulty in cleaning up motion artifacts in real-world scenarios. The MA task showed that the sensors could clearly capture changes in cerebral oxygenation in the dorsolateral prefrontal cortex and demonstrated its generalizability across different subjects. However, we remain concerned about whether this generalizability will hold when switching to other cognitive tasks, such as the Motor Imagery task commonly used in BCI systems. We also observed that preprocessing data during the acquisition process could significantly enhance data quality. By applying real-time pre-processing techniques such as filtering and artifact removal, we ensure that high-quality signals are recorded and stored. This approach not only improves the overall reliability of the data but also streamlines subsequent analysis stages. Additionally, addressing hair-induced artifacts during data collection is another critical factor that has enabled us to expand the sensor system’s data acquisition area. Therefore, optimizing measurements of cerebral oxygenation under complex paradigms, data cleaning, classification algorithms, and hardware design will be key focus areas for further refining our sensor system.

## 5. Conclusions

This paper described the development of a novel, portable, multichannel fNIRS system, specifically engineered to acquire data from the dorsolateral PFC in normal environments. This modular design not only addresses current challenges, but also exhibits exceptional versatility across a broad spectrum of everyday scenarios. The advent of this wireless, multichannel fNIRS technology marks an improvement over existing wireless approaches, particularly with respect to data purification and the refinement of analytical methodologies.

We compared the hardware performance of our fNIRS system with other similar systems and documented the results in Table 4. Different NIRS systems do not have a unified standard for wavelength selection and detector numbers, which vary depending on the specific system. While increasing the number of wavelengths and detectors can enhance signal precision, it also increases power consumption. Additionally, the SD between optical channels should not be too small; otherwise, detection depth may be insufficient. Therefore, our NIRS system’s configuration is more optimized compared to the other three systems. NIRS signals primarily reflect hemodynamic changes, which are low-frequency components. Hence, a sampling rate of 16 Hz is appropriate. The 19-bit ADC provides higher precision than the other systems. Furthermore, the other metrics in Table 4 also place our system in the mid to upper range.

The proposed system integrates a sophisticated noise-reduction framework underpinned by ALCC principles, which is remarkably effective in mitigating a wide array of environmental light interference. The fNIRS signal frequency is inherently low, obviating the need for an excessively high sampling rate. Given the low-frequency characteristics of fNIRS signals, the chosen modest sampling rate of 16 Hz emerges as a judicious balance between efficiency and precision, while the 19-bit ADC resolution of our system significantly enhances the accuracy of signal detection. Each sensor array is energized by a 280 mAh lithium battery, conferring the system with a minimum operational autonomy of 4.5 h. The system’s robustness was tested by evaluating its sensitivity to variations in cerebral blood oxygenation levels, and the results confirmed its high reliability.

To verify the overall SNR of the proposed system, we tested the sensor’s performance under normal acquisition conditions. Subsequently, we conducted a cuff test to validate the reliability of the sensor by comparing it with commercially available single-channel devices. The sensor showed sensitive changes in response to blood oxygen variations, providing a reliable experimental foundation for subsequent tests.

Following the acquisition of multichannel data from the dorsolateral PFC, we meticulously analyzed the dataset to unpack the differentiated responses to attentional stimuli observed across cerebral hemispheres. To evaluate the effectiveness of our fNIRS system within an attention assessment framework, we adopted the MA paradigm. The primary goal of this experiment was to verify that the sensor could detect specific changes in blood oxygen levels following a change in attention. Hence, we designed a simple task to simulate a clear variation in attention load and observe the corresponding changes in blood oxygen levels. This allowed us to directly see the changes in blood oxygen levels in response to attention from the data. Subsequent work will focus on refining the algorithms to better capture changes in attention, using more frequent variations in attention loads to test the system’s accuracy. This could extend the application of our system to various fields such as medical rehabilitation.

## Figures and Tables

**Table 1:** Details of experimental subjects.

Subject	Gender (M/F)	Age (y)	Height (cm)	Weight (kg)	Systolic (mmHg)	Diastolic (mmHg)
S0	M	26	172	65	125	77
S1	M	26	199	108	121	80
S2	M	30	170	90	144	119
S3	F	24	173	78	122	76
S4	M	27	170	68	108	71
S5	M	27	172	81	101	70

**Table 2:** Features of Ch1 quantifying discrimination performance between rest and task sections of MA test.

	ΔHb Max	ΔHb Min	ΔHb Avg	ΔHbO Max	ΔHbO Min	ΔHbO Avg
REST PART I	18.73	7.71	13.86	−32.70	−45.95	−38.65
TASK PART	30.96	4.73	22.64	−13.48	−42.82	−26.71
REST PART II	11.62	4.96	8.02	−24.04	−42.95	−32.84

**Table 3:** Features of Ch2 quantifying discrimination performance between rest and task sections of MA test.

	ΔHb Max	ΔHb Min	ΔHb Avg	ΔHbO Max	ΔHbO Min	ΔHbO Avg
REST PART I	8.22	−5.72	1.49	−1.91	−24.18	−13.26
TASK PART	12.65	−10.71	5.33	−2.42	−41.32	−21.78
REST PART II	−3.40	−12.05	−6.82	−3.17	−20.22	−10.08

**Table 4:** Comparison between our work and three commercial NIRS systems.

	Our Work	Paper [32]	Paper [31]	Paper [33]
Wavlength (nm)	660, 850	730, 805, 850	760, 870	775, 810, 850
Detector Number	1	1	2	4
SD Distance (mm)	18.15	25	40	25
Sampling Rate (Hz)	16	1000	100	10
ADC Resolution	19	16	12	12
Battery Life (h)	4.5	4	4	1
Channel Number	8	4	4	1
SNR (dB)	58	66	37	49

## Data Availability

Data are contained within the article.
